# The slow transformation of the collectivist education of child refugees from Greece in socialist Czechoslovakia

**DOI:** 10.1177/17506980251413210

**Published:** 2026-04-14

**Authors:** Kateřina Králová, Nikola Tohma, Jessie Barton Hronešová

**Affiliations:** 1 Institute of Ethnology of the Czech Academy of Sciences, Czech Republic; 2 Masaryk Institute and Archives of the Czech Academy of Sciences, Czech Republic; 3 University College London, UK

**Keywords:** care system, child refugees, children’s homes, collective education, slow memory, state socialism

## Abstract

Using a Slow Memory framework, which foregrounds sustained, reflective engagement with the past, this article examines the evolving institutional care provided to child refugees from the Greek Civil War (1946–1949) in socialist Czechoslovakia. Rather than interpreting their experiences primarily through trauma or rupture, the analysis focuses on the long-term everyday realities of life in childcare institutions, recalling both supportive and constraining aspects as remembered by the children and shaped by state authorities. Although these institutions were formally aligned with the post-1948 communist project of collectivist education, the study reveals considerable institutional inertia as traditional educational practices coexisted with emerging ideologized forms of socialization. Tracing how a temporary humanitarian intervention developed into a durable system of care and control, the article shows that the children’s lives were shaped not only by political agendas but also by improvisation, routine, resilience, and enduring peer solidarity.

## Introduction

Now I was standing in front of the Mikulov railway station, trembling and unable to take a single step. . . . Gritting my teeth, I slowly made my way to the large gate that opened into my past, inching forward like a snail. . . . I know it was foolish. When the train arrived, I was just a small child and remembered nothing of that arrival. (Agathonikiadis, 2019: 150–151)

This is how George Agathonikiadis, one of the approximately 12,000 Greek Civil War refugees in post-1948 state-socialist Czechoslovakia, recalled his return to Mikulov in his 2019 autobiographical novel. When he arrived 70 years earlier, George was only 2 years old—far too young to remember the journey that brought him there. Yet, fragments of his childhood, spent in a state-run children’s home without his family, have gradually resurfaced, slowly piecing together the story of a prolonged stay in a foreign country where most Greek and Macedonian refugees from Greece clung to the hope that they would soon be repatriated (Agathonikiadis, 2019).

The experience of Greek Civil War refugees both in Czechoslovakia and elsewhere in the so-called “Eastern Bloc” countries (see especially Fokas, 2023; Scutaru, 2023; Tohma and Reinke, 2024; Tsekou, 2010, 2013), and children such as George, is deeply entwined with the slow and complex process of institutional care, education, and integration following their displacement after the Greek Civil War (1946–1949). What the Communist Party of Greece (KKE), representing the refugees, and its allies in the Eastern Bloc initially envisioned as a temporary measure gradually evolved into an extended and unanticipated transition, marked by bureaucratic inertia, institutional dependencies, and shifting legacies. As we document in this article, the displaced children in the *ad hoc* established children’s homes remember the long and complex arc of institutional care, education, and social integration in the 1950s as a slow process of life-shaping interactions.

We explore the children’s remembering at the backdrop of the shifting institutional landscape of postwar Czechoslovakia. While rooted in the Czech system—known for its long tradition of progressive policies, high literacy, well-established school networks and teacher-training institutes reaching back to the nineteenth century (Kasper, 2022; Kasper and Kasperová, 2015; Zounek et al., 2017)—the care for child refugees was reconfigured in the postwar years to align with the communist reform but also with the KKE’s demands for Greek “patriotic” education suitable for the entire Eastern Bloc (Bontila, 2004: 99–121; cf. Qualls, 2020: 63–77). As children adapted to life in state-run homes, the communist state simultaneously introduced innovative policies and institutional frameworks, gradually embedding new expectations for the future. In this sense, the lives of child refugees became entangled in what Reinhart Koselleck (1985) calls the “temporal horizon” of a new political regime—where past experiences, present conditions, and imagined futures were being slowly recalibrated.

Drawing on personal accounts, such as oral-history interviews and memoirs, and institutional records that provide glimpses of institutional memory shifts, this article explores how this transformation unfolded over time and how the then-children reflect on its meaning decades later. Applying the Slow Memory framework (Wüstenberg, 2023) that emphasizes sustained and reflective engagement with lived experience over time, the analysis specifically relies on the personal memories of former child refugees from the Greek Civil War, including two women. From the collection stored in the *Memory of Nation* archive,^1^ the authors selected five interviews that closely focused on the experience of children’s homes. Additional accounts come from published memoirs of Greek male refugees (Agathonikiadis, 2019; Karadzos, 2004; Škoklev, 2018). We also rely on secondary literature and archival material to document changes in institutional memories. It should be noted that there is a paucity of female refugees’ reflections on their stay in Czechoslovakia, which is why we included two in particular. These life stories and in-depth interviews with adult child refugees from Greece respond to the slow, cumulative engagements with the process of institutional care transformation.^2^

In what follows, we argue that the experiences of Greek Civil War child refugees in Czechoslovakia are best understood through the lens of Slow Memory as a form of more natural, relational, and organic interpretation of past processes and remembering that emerges over time through routine practices, enduring social ties, and the long-term imprint of institutional life. Approaching memory as the result of a long-lasting and slow process means focusing less on singular “impact events” (Fuchs, 2012)—transformative, often traumatic occurrences that figure prominently in collective memory—and more by the long-term workings of institutions, in both the negative and the, perhaps less expected, positive sense. Such “slow memories,” as Wüstenberg (2023) notes, are “often without clear location” or a single defining moment, yet are “no less important or transformative for human (and nonhuman) lives” (p. 60). Rather than analyzing refugees’ lives through isolated mnemonic turning points, traumas, and ruptures—such as departure from Greece or other discrete episodes—we argue that their childhood memories are better understood as slow-forming, rather eventless processes, difficult to locate in time: the gradual and layered effect of daily routines, mundane practices, and habits embedded within institutional settings.

While social, collective but also cultural memories are created as amalgams of social and cultural frameworks (Assman, 2011; Olick, 1999) and everyday memories are often fragmented, affective, and tied to lived experiences (Connerton, 1989), the Slow Memory approach has a different task. It attends to continuity, durable practices, long-term caring relationships, and the everyday ways in which people make sense of the past as part of ordinary life. As such, Slow Memory foregrounds the ways in which gradual, institutionally embedded routines are retrospectively reworked into memories. Moreover, Slow Memory also directs attention to the ambivalence of the everyday practices when seemingly nothing happens and their accumulation over time. Where everyday memories narrate instances of practices, the Slow Memory approach puts them in a wider context. The incremental, often contradictory processes, such as schooling, bureaucracy, waiting, can be remembered as both enabling and constraining. In this sense, it provides a lens for analyzing how exile and reintegration are recalled not through singular turning points or instances of daily interactions but through the sedimentation of lived practices, feelings, and emotions, thereby better capturing the complex temporalities of refugee memory.

In our case, what began as a temporary humanitarian intervention transformed into a durable system of care and control, wherein the socialist state’s ideological and bureaucratic frameworks both enabled and constrained the lives of child refugees. Over time, the legacies of the children’s homes became fixed not only in political and institutional histories but also in the layered, affective terrain of memories—where personal recollections mediate between past traumas and continuous improvisation, resilience, and long-lasting peer solidarity. To showcase our argument, we first provide the context for the arrival of child refugees from Greece to Czechoslovakia between 1948 and 1949, outlining the key sociopolitical aspects of their integration, supported by memories of the refugee residents of the children’s homes. The focus then shifts to the evolving institutional landscape of residential childcare in postwar Czechoslovakia, emphasizing the role of the Czechoslovak Red Cross (CSRC) and educational institutions as important sources for administering memories of displacement and care for the Greek Civil War child refugees. We further explore how educational institutions participated in these processes, gradually reshaping the frameworks through which care was conceptualized and delivered. Personal accounts are again employed to illustrate how these transformations felt on the ground. In the final section, we analyze socialist children’s homes as hybrid institutions—spaces of care, education, and memory-making—highlighting both their transformative ambitions and the limitations of this effect.

## Key aspects of postwar socialist residential childcare

The Greek Civil War was still ongoing when Czechoslovakia began receiving the first child refugees. Between April 1948 and December 1949, about 5185 children arrived in the country, followed by 6910 adults between August 1949 and January 1950 (Botu and Konečný, 2005: 28–32; Hradečný, 2000: 24–37; Králová and Tsivos, 2012: 42). Whether orphaned, alone, or with their families, most of the children spent extended periods of time—often their entire childhood—in residential care. It was also a common practice in Czechoslovakia to move them between multiple facilities, losing their connections with friends, educators, and even families. This was caused more by capacity needs than by any political events occurring at the time.

One illustrative case is that of Georgia Zerva, a Modern Greek philologist and author of several Greek language textbooks. Born in 1946 to Greek communist partisans, her pregnant mother left for Buljkes, an autonomous KKE-run commune in Yugoslavia, which served as a haven for communist fighters and their families from Greece (Králová, 2016; Ristović, 2006, 2012; Rolandi, 2025). However, as political tensions between Joseph Stalin and the Yugoslav leader Josip Broz Tito intensified in 1948, resulting in the split between Yugoslavia and the USSR that summer, the settlement was dissolved, and its residents dispersed across Soviet satellites. Georgia’s family was among those relocated, and she ultimately found herself in Czechoslovakia. Separated from her parents for much of her childhood—and as a single child—Georgia passed through nine different children’s homes, leaving the last one for a boarding school at the age of 15. Building on the collective identity forged among Greek Civil War child refugees in residential care, Georgia Zerva evokes the enduring community of memory shaped over decades, using “we” to articulate the shared experience of herself and her peers:

When we were very little, all that moving around was extremely demanding. You can imagine—my mother and stepfather settled in Bohumín near Ostrava [in Moravia], and we, for example, were in South Bohemia near Nové Hrady. . . . It was impossible for them to travel such a long distance, so as small children, we spent the holidays in the children’s homes. (Personal interview with Zerva, 2010)

The socialist transformation of post-1948 Czechoslovakia had a far-reaching and lasting impact on social care and education, leveraging established school networks and teachers’ training but leading to increased ideological control. Previously provided mostly by churches, private organizations, and public associations, in the aftermath of World War II, residential childcare was restructured as a state service, initially targeting orphaned, abandoned, and displaced children within Czechoslovakia (Act No. 48/1947: 25). As children’s homes for Greek child refugees began to emerge in 1948, the national system of institutional care was undergoing reform, making the integration of child refugees a part of a broader, evolving process.

From today’s perspective, these children’s homes became both individual and collective sites of memory, in the sense described by Pierre Nora (1996), but also through the spatial conceptualization of the past as a series of shifting actions, entangled temporalities, and cross-scalar connections tied to locations in the time and space (Shaw, 2010). Established in dozens of buildings, often consisting of confiscated aristocratic or Czechoslovak German property, recreational and spa facilities (Botu and Konečný, 2005), these homes, as silent witnesses to displacement and nationalization, continued to carry hidden historical legacies that had been largely unacknowledged by the state given their rather slow transformation. Yet, the Greek Civil War refugees, coming from quite different cultural settings, were much aware of the distinctive character and atmosphere of these locations, with many lost and hidden memories within them.

The educator and former chairman of the Greek Committee for Aid to Children (EVOP) in Czechoslovakia, Lysimachos Papadopulos, spent several years visiting the children’s homes. In his book, he recalls Budišov affectionately, as a “beautiful chateau in a stunning setting.” The children’s home in Velké Heraltice was, according to him, “surrounded by a large park with centuries-old trees. The interiors are magnificent, and some rooms are decorated with rare wall paintings.” He remembers Šilheřovice as located “in the middle of an extensive park inhabited by game animals. Every year, aristocrats had gathered there for a hunt, after which they had retreated to a small hunting lodge, where they were served delicacies prepared from the game they had caught” (Papadopulos, 1999: 83–84). His account thus provides a romantically framed glimpse onto the setting of these homes.

Among the main reasons for the placement of the Greek Civil War child refugees into such homes were the economic vulnerability of their refugee parents, their lack of Czech knowledge, and a low capacity to navigate the socially and culturally distinct environment. Equally significant was the prevailing belief that socialist state administration and care organizations were superior to family placements. Rooted in the state’s social engineering ambitions, these institutions were seen as a means of constructing an entirely new society through presumably scientific methods, building upon knowledge of nutrition, socialization, and children’s psychology at that time (Henschel, 2016), and thus creating new repositories of memories.

The children’s homes also functioned as practical tools for the wider socialization and integration of Greek Civil War child refugees. Even during economic decline and political instability in the aftermath of World War II and the emerging communist rule, the state homes provided material benefits, skills, resources for professional success, and knowledge that many parents, given their circumstances, were unable to offer (Tohma, 2025). When reflecting on their childhood experiences, many former refugees acknowledged the advantages of this institutional upbringing, particularly in terms of their preparation for adult life, even as they grappled with the emotional detachment from their families. With a sense of understanding, Sotiris Joanidis, who graduated from a forestry school, remembers the situation of his parents: “my father wouldn’t have been able to feed us, there were no clothes, nothing. In the homes, we were given food five times a day. Czechoslovakia took care of us” (cited in Poláková, 2020: 121; see also interview with Joanidis, 2010).

Georgios Karadzos, who also grew up in children’s homes, attributes his professional career as a surgeon and head of department to the opportunities offered to him by the host country: “The fact that we ended up in Czechoslovakia—I still consider it an excellent choice for further development. Because the countries around us were not as advanced, not as well-equipped in terms of engineering, universities, education, and so on.” He further compared the child refugees with their peers who had remained in their homeland in northern Greece: “They told us, ‘You, kids, will go there and study,’ and all of that turned out to be true. . . . our peers [in Greece] had nothing. They didn’t even finish primary school” (personal interview with Karadzos, 2010). Such comparisons may as well function as a retrospective coping strategy to deal with loss and uprooting (Batcho, 2013). However, such comparisons also shaped how former child refugees justified and reframed their separation: transforming it slowly from a painful rupture into a lasting memory of opportunity and personal advancement.

## Institutional landscape of care and aid to the Greek Civil War child refugees

The children’s memories need to be embedded in the complex and evolving institutional setting of the postwar Czechoslovak state. As the children arrived in Czechoslovakia, they became entangled in the state’s own institutional challenges, which often affected their experiences and opportunities. Between 1948 and 1951, the care was managed by the Czechoslovak-Greek Society, a state-funded and only non-governmental organization, directly linked to the Ministry of Labour and Social Care. In 1949, the oversight shifted to the Regional National Committees (NA-147-580, 9 December 1950: 2–3), and from 1952 to 1954, the CSRC took charge. Importantly, the 1953 School Education Act subordinated residential care facilities to the state schooling system (Act No. 31/1953: 14). Recognizing the long-term impact of residential care on children’s development, state authorities sought to use these homes as instruments for new educational strategies aimed at transforming Czechoslovak society (Štejgerle, 1955: 5). The legal change in the institutional framework gradually turned the notion of how the children’s homes were perceived. They were no longer merely places of care for child refugees of the Greek Civil War, but unfolded as sites of general, social, and political education, with a strong emphasis on communist ideology.

By 1955, infant care moved under the Ministry of Health, and care for children aged 3 to 15 fell under the Ministry of Education. While state control improved material conditions and education, constant administrative changes caused organizational problems, further exacerbated by the lack of pedagogical training among the personnel (Tohma, 2025). Inefficiencies and variations in resources, regional oversight, and capacities meant that some homes needed more time to implement reforms than others. Moreover, the sheer scale and novelty of the project—caring for thousands of traumatized, multilingual, and culturally distinct children until their adulthood—was ambitious and potentially overwhelming for the system. As a result, the intended transformative role of institutional care was fulfilled only partially, more often limiting itself to the provision of the basic needs rather than investigating radically new educational methods.

The transformation of residential care for child refugees was shaped by what social scientists in politics and economy would call today both “path dependency” and “institutional inertia”—structural processes that resist abrupt change, escape the attention of, and are hardly remembered by the former residents (Beyer, 2025; Cornwell, 2015; David, 2020; Faghih and Samadi, 2024). Such gradual developments can thus be well captured by the Slow Memory framework. In practice, this meant that Soviet pedagogical principles following Anton S. Makarenko (1888–1939) were only gradually embedded within the elements of the well-established Czechoslovak childcare traditions of the interwar period (Makarenko, 1952; see also Potočárová, 2021).

The CSRC played a key role in sustaining aspects of a more historically rooted tradition of care for the refugee children although it also had to conform to the evolving socialist framework. Restored after World War II, the organization became principally state-controlled, with its leadership compliant with the ruling Czechoslovak Communist Party (KSČ) (CSRC, 1955). Although clearly aligned with communist principles of internationalism, world peace, and equality regardless of their ethnic origin (CSRC, 1958), its grassroots initiatives remained focused on health care, hygiene, and practical welfare respecting its original spirit and maintaining a certain independence from the state (Hachmeister, 2019). After the war, the CSRC continued to function as a state organization, drawing on its earlier humanistic mission. Motifs of mutual aid and cross-border understanding—prominent in its prewar activities—were now reframed as consistent with the principles of socialist internationalism (Brázdová, 1956: 2–3). However, this continuity was selectively constructed.

In the 1956 booklet compiled by Zdeňka Brázdová, published in several languages, the legacy of Alice Masaryková (1919–1938), the organization’s first director and daughter of Czechoslovakia’s first president was entirely absent (Berglund, 2011; Lovčí, 2020). Instead, the CSRC traced its ideological lineage to Jan Ámos Komenský (Comenius) (1592–1670), reinterpreting his educational philosophy and critique of human selfishness as an endorsement of collectivist values. In doing so, the organization actively reshaped the public memory of its own past. Tellingly, when quoting from Comenius’ *Great Didactic (Didactica Magna)*, religious references were deliberately omitted to comply with the communist ideology (see Brázdová, 1956: 1). A similar pattern emerged in the portrayal of the CSRC’s efforts in combating child poverty before the war. Although the organization’s previous achievements in reducing poverty were recognized, the story was reshaped to support the official position that child poverty no longer existed in communist Czechoslovakia. This way, it CSRC was transformed into a predominantly health-focused institution and “the guardian of the national health” (Brázdová, 1956: 4–5). Over time, this ideological, yet inconspicuous memory transformation of what CSRC stood for effectively merged national and internationalist agendas, reinforcing the broader communist worldview both within the Greek refugee community and the general public.

## Shaping frameworks for educational transformation

Beyond the Czechoslovakia-specific factors, the children’s homes were embedded in a wider context of the communist narrative of progress and principles of Soviet pedagogy as the model for recreating evolving socialist societies. Children’s homes were “intended to prepare children for work, to cultivate in them love and a communist relationship towards labor, as well as the endeavor to work for the good of the country” (Šastov, 1954: 3). This way, they were to contribute to the creation of “versatile” people, positioning Marxism-Leninism as a major source of the educational approach (Šastov, 1954: 4). At its core, the system upheld a belief in modernity, particularly the application of advanced industrial technologies and the novel methods of agrotechnics developed by Ivan Michurin (1855–1935), symbolizing the capacity of human will and scientific planning to reshape nature itself envisioned as a horizon of the future (Šastov, 1954: 5).

Children’s homes were fundamentally designed to prepare children for work by developing their skills and shaping their moral and psychological outlook, closely linked to collectivism and labor education. According to Makarenko (1952), “It is only by participating in common work that a human can form a right moral relation to other people—to feel love and friendship for every working person and dislike and loathing for the idler who shuns work” (p. 48). Without parental guidance, educators facilitated exposure to everyday life through well-remembered visits to factories and collective farms, as well as voluntary work, reinforcing both the economic value and social significance of the children’s homes. Children’s labor was thus seen as a vital tool of ideological and political education, designed to instill respect for socialism, common property, and discipline (Šastov, 1954: 5–9).

Daily activities and annual events, notably the organized summer youth brigades, formed an important part of the children’s mnemonic fabric. As one former Greek Civil War child resident, Kosta Škoklev (2018) recalls, “During the harvest we loaded sheaves onto cars or tractors, and we picked fruit . . .. We were paid for the work, but we used to say that the state gives us a lot, that it provides us with many benefits” (p. 52). Georgios Karadzos agreed, adding that for him as a young teenager a regular summer placement in agriculture was not only financially profitable but also offered a sense of personal freedom: “In the summer, we helped local farmers. They even gave us a bit of money, bread with lard and cracklings. And there was this sense of freedom—no one bothered us” (personal interview with Karadzos, 2010). Although it was forbidden for the homes to use children’s work for economic benefit (Štejgerle, 1955: 148), another former resident, Martha Elefteriadu, remembers that she did not mind working as a child: “Mostly, we worked in the garden. I really enjoyed it.” And she added that “even so, we had plenty of time for mischief, for wandering through the woods and parks” (personal interview with Elefteriadu, 2010). Rather than eventful, these memories were a combination of everyday and lived memories that were embedded in a wider contextual life trajectory across time.

Diverging from the stricter Soviet model, socialist Czechoslovak education aspired to create “the people of a new type” (Štejgerle, 1955: 14) but also addressed children’s needs more attentively. While grounded in socialist ideology, the Czechoslovak system maintained a more flexible stance on the role of family upbringing, even as that role was gradually redefined. Early on, the socialist state recognized the family as an institution but subordinated it to national interests, prioritizing parental labor—especially for women—over direct childcare (Štejgerle, 1955: 14). In the 1950s, residential care was often viewed as superior to family upbringing, promoting personal development and eliminating “narrow family selfishness” (Stuchlý and Rottová, 1950: 3–4). By the 1960s, however, public and social institutions were seen as necessary to “save” the family, suggesting a shift in state policy (Michal, 1963: 8).^3^ This transformation signaled a new phase in the relationship between the state and the private sphere—one that recognized the limits of institutional care and sought to integrate family life into the socialist project rather than displace it. Yet, the absence of these processes has escaped the memory of the former residents who never reflected on this political relationship between the state and family.

As such, children’s homes for Greek Civil War refugees were primarily meant to substitute for family care due to socioeconomic, health, and educational reasons, with the benefits of sustaining contact between children and their parents—although assessed on an individual basis (Štejgerle, 1955: 5, 149–150). Nevertheless, state facilities for parentless and abandoned children—called children’s homes rather than orphanages in post-1948 Czechoslovakia—continued to carry negative connotations. In broader society and collective memory, they were often linked to squalid, Dickensian conditions of care (Waters and Merchant, 2015). Already during the state socialism, institutional childcare also attracted strong criticism from some leading figures in children’s psychology, such as Zdeněk Matějček, who advocated for more emotionally supportive and family-like environments (Langmeier and Matějček, 1970; Matějček, 1969; Matějček and Koluchová, 2002). Yet, the facilities for Greek Civil War child refugees stood apart, gaining a reputation for genuine solidarity and hospitality as recalled by their former child residents (Králová and Tsivos, 2012; Tohma and Reinke, 2024). The special bond among the residents of the children’s homes is evident even decades later. Capturing the uniqueness of that connection through daily habits and encounters, Georgia Zerva recalls that

all the children who grew up in the children’s homes—even those I didn’t personally know [were friends] . . . There are these brotherly, sibling-like relationships. That means if I saw someone being wronged, I would do everything I could to protect them. (Personal interview, 2010)

In the early 1950s, as parents of the child refugees started arriving in Czechoslovakia, the country’s authorities faced growing numbers of requests to return children to family care (e.g. MZA-9-22, 21 November 1950). The children’s homes often resisted. Parents were perceived less as a source of emotional support for the children and more as a disruption to the structured environment of the homes. The state considered parental visits—typically escorted and overseen by administrative staff—costly and logistically burdensome. Given the geographical distance between parents and their children within the country, the sporadic visits that are only vaguely remembered by the children often led to forced, unanticipated overnight stays in already overcrowded facilities, which the staff struggled to tolerate. Instead, some refugee mothers sought employment in the homes to stay close to their children, leading to what was perceived as overemployment, reaching a ratio of one to ten of caregivers to children or more (NA-147-580, 24 March 1950). Although their presence was officially criticized for inefficiency, it was simultaneously promoted for political advantage: a “guarantee” that the children were being raised in a Greek cultural environment (NA-147-580, 13 September 1950).

Although the transformation of Czechoslovak education was proclaimed a revolutionary success, state experts in social and political work with children continued to critique its lasting limitations, calling for improved pedagogical methods, better evaluation procedures, and enhanced knowledge-sharing across institutions. Yet, systemic change was hindered by entrenched bureaucratic routines and established ideological frameworks. The principle of “socialist humanism” (Štejgerle, 1955: 6–14)—a reformist Marxist ideology that emphasized individual dignity, ethical socialism, and the development of the person within a supportive collective—served only as a rhetorical guidepost for these reforms, supporting the reintegration of children into society and emphasizing the collective as a political and moral space.

In parallel, Greek and Czechoslovak authorities were keen to ensure the continuity of Greek national identity to prepare the children for their eventual return to Greece. Teachers from Greece initially taught within the homes in the children’s native languages (Greek, Macedonian) while fostering practical knowledge, social awareness, and respect for work (MZA-9-22, Rules for teachers). For example, as regards language classes, former child residents note that the Greek language was taught consistently in the homes, and in Sobotín, Macedonian pupils also had lessons in Macedonian, alongside a reduced number of Greek language classes: “There, we were divided by age and we simply started going to school . . . so the Greek children had four hours of Greek daily, the Macedonian children had Macedonian like that, and in addition, two hours of Greek” (interview with Miovský, 2011). On top of that, as Zerva recalls,

in the children’s home, we also had the benefit that every week, either on Friday or Saturday, the head educator—a Greek, who was very educated and a former teacher—would always present the news in Greek. So we actually knew what was happening in Greece. (Personal interview, 2010)

Over time, instruction shifted increasingly to Czech, and from around 1952/1953, children were gradually integrated into Czech schools. Older pupils were funneled into vocational training as soon as possible, particularly to industrial fields, areas thought to be lacking expertise in Greece but also beneficial for the intended communist transformation of Greece (NA-147-580, 13 September 1950). Grounded in Makarenko’s legacy, collectivism was elevated as the foundation of moral education, subordinating individual interests to those of the group. This ethos was further extended into the realm of socialist internationalism, promoted as the highest form of true patriotism. The institutional care of Greek Civil War child refugees—symbolic of these trends—was held up as a model embodiment of these ideals.

Yet, the child refugees’ memory of this care, largely omitted within Czech population but well preserved in institutional narratives and retrospective accounts of the child residents, reveals the persistence of structural contradictions, where lofty ideological goals met the stubborn realities of institutional constraints. It also uncovers a complex process of remembering of daily and mundane activities in a wider context that go beyond everyday memories. Seen through the lens of “Slow Memory” (Wüstenberg, 2023), such protracted policies left no clear events to crystallize memory and framed daily activities in larger experiences. They worked incrementally, embedding loss, inefficiencies, and ambivalence into the everyday lives of refugee children. For the child refugees, memories of this period emerge not as one defining trauma of loss and rupture but as the slow sedimentation of institutional practices and habits in the wider context of long waiting, postponed reunions, and the normalization of separation, which shaped how these individuals narrated belonging and family.

## Blended laboratories of care, education, and repositories of slow memories

For many former child refugees from Greece, children’s homes have *de facto* become sites, realms, and milieux of memory (Nora, 1996; Wüstenberg, 2023), preserving personal and collective narratives shaped by both institutional discipline and solidarity, order, and affection. These homes were embedded in the politics of time and temporality, reflecting a fluid tension between their proclaimed temporary status and the long-term imprint they left on individuals and institutional memory. In keeping with the ethos of internationalist solidarity, the children’s homes were expected to ‘compensate for family upbringing, to develop the children’s skills, and educate them to become democratic citizens of their country’ (MZA-9-22 Home rules for age 6–14: 1). This vision reflected a transnational orientation aligned with the global communist movement and, at the same time, served as a site of political experimentation, imagining new models of care, identity, and belonging aligned with socialist ideals. To realize the communist ambitions, the homes operated through a structured division of labor. However, what began as separated refugee-specific treatment gradually transitioned toward integration and normalization of the children from Greece within the Czechoslovak educational system. For the child refugees, this also meant navigating and blending two distinct social cultures and realities—their Greek heritage and language on the one hand, and the norms, language, and expectations of socialist Czechoslovakia on the other. As such, they facilitated a distinct form of mnemonic communities, as we describe below.

The Czech–Greek political dimension of the children’s homes revealed critical vulnerabilities. Ideological oversight was meant to be enforced through party organizations and appointed instructors—both often absent or difficult to staff. Ethnic and political tensions, especially between Greeks and Macedonians after the Tito-Stalin split in 1948 and amid the 1956 purges in the exiled KKE, further destabilized internal cohesion. Compounding the issue, investigations exposed the “infiltration” of politically unreliable Czechoslovak personnel, including those linked to World War II collaboration or the liberal legacy of interwar Czechoslovakia. Breaches of ideological discipline were documented, such as a cook attempting to flee across the border and a caretaker displaying religious imagery (NA-147-580, 24 March 1952: 2–3). These patterns reflected path dependency, where historical affiliations and loyalties continued to shape the newly introduced social and political climate.

The children’s homes nonetheless demonstrated efforts to promote relatively progressive or modern approaches to care, less prevalent and commonly implemented within family settings. While the ideal child was envisioned as obedient, well mannered, and orderly, these attributes were instilled through praise, patience, and non-coercive discipline rather than corporal punishment (as was then a common practice in many families at that time). Public praise was encouraged as the main motivational tool, while punishment was formally limited to verbal reprimands or exclusion from treats and leisure. Corporal punishment and deprivation of basic needs by staff were strictly forbidden and punishable by dismissal in the children’s homes (MZA-9-22 Rules for teachers; Home rules for age 6–14; cf. Štejgerle, 1955: 50–53). Regardless, according to Zerva, the beating of children in the homes where she used to stay was persistent, although she does not have any recollections of it affecting her personally (personal interview, 2010). In contrast, Karadzos vividly recalls that a Greek teacher used corporal punishment on him after he opposed the teacher’s will, leaving him “with an extremely sore body, bruises, and a bloody nose.” Yet, somehow apologetically, Karadzos (2004) claims that this was “the first and last beating” he had ever received from his caregivers (pp. 28–29).

The educational system in the children’s homes also encouraged children’s agency. A “self-management” framework was introduced, allowing children to organize their after-school activities, take responsibility for rules, and elect peer committees modeled on popular governance (MZA-9-22 Home rules for age 6–14). This structure aimed to cultivate a long-lasting sense of responsibility, equality, and institutional identification. Younger children remember that although they were not as targeted by ideology as their older peers, they also followed structured daily routines emphasizing hygiene, nutrition, independence, and outdoor play. Their care was increasingly professionalized and results-oriented: in 1950, improved nutrition led to average weight gains of 5 kg across age groups (MZA-9-22 Home rules for age 2–6; MZA-9-22, 4 March 1950; and NA-147-580, 13 September 1950). As Soviet manuals framed hands-on activities as both pedagogical and political, shaping children into well-rounded socialist citizens (Šastov, 1954: 9), children’s homes were filled with books, new toys, radios, projectors, as well as Greek and communist symbols, such as images of the Acropolis of Athens and the Red Square in Moscow (MZA-9-22 List; NA-147-580, 13 September 1950). Education thus followed the principle of “polytechnization,” blending learning with labor and social life (Štejgerle, 1955: 30–32, 35). Children engaged in gardening, chores, and, with time and growing openness beyond the isolation of children’s homes, also in cultural activities that connected them to broader society (Štejgerle, 1955: 145–147). However, while soon well versed in ideological vocabulary, as the former pupil Kosta Škoklev recalls, naming everyday objects, such as a bread-roll (staple in Czechoslovakia, yet uncommon in Greece), proved difficult due to their lack of Czech language skills and the unfamiliarity of the new environment (Škoklev, 2018: 29–30).

In spite of the ruptures of war, displacement, and institutionalization, the children who passed through the socialist children’s homes found ways to navigate and ultimately cope with their complex realities. Many cultivated deep bonds with peers, forming enduring networks of fictive kinship that offered emotional support in the absence of family. These relationships, together with the structured yet educationally productive and nurturing environment of the homes, enabled the children not only to survive but actively reconstruct a sense of belonging and purpose. Through shared routines, collective experiences, and a pedagogy that—despite its ideological aims—promoted responsibility, cooperation, and resilience, they developed a communal identity that transcended national and ideological boundaries (Danforth and Van Boeschoten, 2012; Králová and Hofmeisterová, 2020). This community of memory provided a protective space for reinterpreting the war trauma, asserting agency, and resisting marginalization in their new homeland. The children’s homes thus evolved from mere sites of care into sites of memory, spaces where childhood trauma was rearticulated through collective remembrance and mutual support, with memories anchored less in moments of rupture than in the continuity of everyday routines and practices, resonating with the conceptual framework of Slow Memory.

## Conclusion

More than just temporary havens for displaced children, children’s institutional homes for Greek refugees in socialist Czechoslovakia functioned as intricate laboratories of care, education, and ideological formation. As experimental spaces of socialist transformation, they intertwined internationalist solidarity with efforts to compensate for disrupted family structures, cultivating children into citizens aligned with socialist ideals. They became sites of lasting remembrance, where protective ideals often produced harm, dysfunction lingered despite reforms, and childhood memories were infused with a sense of lost belonging. However, these homes also became formative sites where order met improvisation, and where socialist ideals clashed with the realities of everyday care, leaving a lasting imprint on the lives they shaped. As such, the story of Greek child refugees in Czechoslovakia unfolds not through dramatic ruptures but via a slow process of remembering daily experiences, acts, habits, and routines. Initially framed as a temporary humanitarian response, the care provided to these children gradually morphed into a durable system of institutionalization.

This slow transformation was underpinned by bureaucratic routines and ideological imperatives that prioritized political cohesion over individual needs. Over time, though, the temporary turned permanent, embedding child refugees into the relatively novel socialist project while complicating their paths to familial reintegration and cultural continuity. Central to this dynamic was institutional inertia—the persistent resistance to structural reforms despite evident shortcomings such as the lack of skills of the staff—but also path dependency as the continuity of previous humanistic legacies, as embodied in the CSRC. While there were numerous critiques regarding inadequate staffing, insufficient pedagogical training, and ideological inconsistencies, the children’s homes rarely adapted in ways that addressed these problems. These homes thus became emblematic of a system both committed to and constrained by its own ideological frameworks.

At the same time, these institutions became new and unique realms of memory—dense with emotion, contradiction, and remembering of the past. Over time, former child residents began to reconstruct their pasts, not only as stories of displacement and ideological education but also as spaces of community, affection, and even joy. Memories of playtime, toys, songs, chores, and shared moments blended with the recollection of institutional rules, disciplines, and emotional absences. These fragments form what has been described as Slow Memory: a process of remembering that unfolds gradually and in a complex web of glimpses of the past across long periods of time, often catalyzed by later-life reflection or symbolic returns to meaningful places. This approach challenges simplistic narratives of either suffering or ideological indoctrination in the children’s homes. Instead, it suggests that memory is negotiated and intimately tied to place and practices across long periods of time. For many former residents, the children’s homes stand as both situational and temporal sites of loss and resilience—spaces where socialist ideals met human improvisation. These memories are not fixed; they shift with time, shaped by broader changes in public discourse, historical scholarship, and personal life trajectories.

The legacy of children’s homes for Greek refugees in Czechoslovakia cannot be reduced to a single narrative or a single type of memory. The memories of former child residents were not uniformly collective, as their experiences differed, yet they were also not purely individual or everyday. Instead, they were shaped in relation to wider political, pedagogical, and institutional settings and influences. These homes functioned simultaneously as laboratories of care and education, spaces of ideological formation, and sites of emotional and social development. Over time, they became repositories of layered memories shaped by personal experience, shifting political priorities, institutional reforms, and the gradual adaptation of socialist pedagogical ideals. Their history reveals the complexity of care under state socialism, the slow and uneven rhythms of institutional change, and the ongoing human effort to find meaning in disrupted childhoods. By considering both structural dynamics and personal recollections, the Slow Memory approach helps us understand how displaced childhoods were lived, negotiated, and remembered across decades. These insights have contemporary relevance for refugee integration policies, highlighting that long-term care must attend not only to material provision but also to the emotional and mnemonic worlds in which children develop and thrive.

## Notes

1. Memory of Nation website: https://www.memoryofnations.eu/en.

2. The interviews were recorded as part of the project “Sixty Years After. Memory of Greek Civil War Refugees in Czechoslovakia, 1949-2009,” available at Memory of Nations, https://www.pametnaroda.cz/cs/sixty-years-after-memory-greek-civil-war-refugees-czechoslovakia-1949-2009.

3. Some Czechoslovak families expressed interest in fostering the newcomer children, but this possibility was ruled out by the authorities in agreement with Greek communist representatives, who favored residential care as a means of preserving the children’s national identity and language skills (MZA-B 280-4531).

## Interviews

Elefteriadu M. Interviewed for *Memory of Nation*, 2 June 2010.

Joanidis S. Interviewed for *Memory of Nation*, 26 June 2010.

Karadzos G. Interviewed for *Memory of Nation*, 22 May 2010.

Miovský K. Interviewed for *Memory of Nation*, 29 June 2011.

Zerva G. Interviewed for *Memory of Nation*, 25 May 2010.

## Archival sources

Act No. 48 of 19 March 1947 on the organization of youth care, April 11, 1947.

Act No. 31 of 24 April 1953 on the school system and teacher education (School Education Act).

### National Archives of the Czech Republic (NA), Prague

NA-147-580: 1261/2 KSČ ÚV 100/3, volume 147, archival unit 580.

-Report on the “Greek Action” by Tomáš Kosta, 24 March 1950.

-Report on the “Greek Action” by Tomáš Kosta, 13 September 1950.

-Report on the “Greek Action” by Tomáš Kosta, 9 December 1950.

-Minutes from the meeting between the representatives of the Czechoslovak Red Cross and the Education Ministry regarding the education of Greek children, 8 January 1952.

-Report on the current situation in Moravian children’s homes by Marie Thelenová (26 February–11 March 1952), 24 March 1952.

-Evaluation of the auxiliary teaching activity of the Education Ministry, July–August 1952.

### Moravian Land Archives in Brno (MZA)

MZA-B 280-4531: B 280 ZNV Brno, box 4531.

-Various letters from Czechoslovak citizens.

MZA-9-22: B 125 KNV Gottwaldov, box 9, inventory number 22.

-Equipment for Greek children, 20 April 1948.

-Monthly report by Mrs. Adámková, Ždánice, February 1950, 4 March 1950.

-Parental visits, Branky (Moravia), 21 November 1950.

-Home rules for Greek children aged 2 to 6.

-Home rules for Greek children aged 6 to 14.

-Healthcare rules for homes of Greek children.

-Rules for educators in homes for pre-school children; rules for educators in homes for schoolchildren.

-Rules for teachers in homes for Greek children.

-Rules for nurses in homes for Greek children.

-List of necessary equipment and tools for education of Greek children.
